# Finding the positive feedback loops underlying multi-stationarity

**DOI:** 10.1186/s12918-015-0164-0

**Published:** 2015-05-28

**Authors:** Elisenda Feliu, Carsten Wiuf

**Affiliations:** Department of Mathematical Sciences, University of Copenhagen, Universitetsparken 5, Copenhagen, Denmark

**Keywords:** Bistability, DSR-graph, Feedback loop, Injectivity, Interaction graph, Reaction network

## Abstract

**Background:**

Bistability is ubiquitous in biological systems. For example, bistability is found in many reaction networks that involve the control and execution of important biological functions, such as signaling processes. Positive feedback loops, composed of species and reactions, are necessary for bistability, and generally for multi-stationarity, to occur. These loops are therefore often used to illustrate and pinpoint the parts of a multi-stationary network that are relevant (‘responsible’) for the observed multi-stationarity. However positive feedback loops are generally abundant in reaction networks but not all of them are important for understanding the network’s dynamics.

**Results:**

We present an automated procedure to determine the relevant positive feedback loops of a multi-stationary reaction network. The procedure only reports the loops that are relevant for multi-stationarity (that is, when broken multi-stationarity disappears) and not all positive feedback loops of the network. We show that the relevant positive feedback loops must be understood in the context of the network (one loop might be relevant for one network, but cannot create multi-stationarity in another). Finally, we demonstrate the procedure by applying it to several examples of signaling processes, including a ubiquitination and an apoptosis network, and to models extracted from the Biomodels database. The procedure is implemented in Maple.

**Conclusions:**

We have developed and implemented an automated procedure to find relevant positive feedback loops in reaction networks. The results of the procedure are useful for interpretation and summary of the network’s dynamics.

**Electronic supplementary material:**

The online version of this article (doi:10.1186/s12918-015-0164-0) contains supplementary material, which is available to authorized users.

## Background

Bistability, and multi-stationarity in general, is ubiquitous in biological systems ranging from biochemical networks to epidemiological and eco-systems [1-4]. It is considered an important biological mechanism for controlling cellular and bacterial behaviour and developmental processes in organisms, and it is closely linked to the idea of the cell as a decision making unit, where a continuous input is converted to an on/off response corresponding to two distinct states of the cell [5,6].

The question of bistability therefore arises naturally in many contexts. Many studies aim to demonstrate that in a given biochemical system, bistability can or cannot occur [2,3,7-10]. There are several methods that can be used to address whether a network is multi-stationarity or not, see for example [11-19] and the references therein. Some of these methods are implemented in the CRNT toolbox [20] or in CoNtRol [21]. More general there has been some interest in formal methods that connect the network structure to the dynamic behaviour of the system, see e.g. [15,22-27].

One qualitative network feature has in particular been linked to multi-stationarity, namely the existence of a *positive feedback loop*. A positive feedback loop consists of a sequence of species such that each species affects the production of another species, either positively or negatively, and such that the number of negative influences is even. The idea of associating positive feedback loops with bistability goes back to Jacob and Monod who introduced it in the context of gene regulatory networks [28]. It was later formalised by Thomas in the form of a conjecture [29], which was finally proved by Soulé [30], see also [31,32].

Soulé considers dynamical systems of the form

(1) $$ \dot{x} = f(x),\qquad x\in \Omega\subseteq \mathbb{R}^{n},  $$

where *x*=*x*(*t*), *x*=(*x*_1_,…,*x*_*n*_) is the vector of species concentrations, $\dot {x}=dx/dt$ is the derivative of *x* with respect to time *t*, and *f* is the so-called species-formation rate function, which specifies the instantaneous change in the concentrations.

The work of Soulé is based on the so-called *interaction graph* [30]. This graph encodes how the variation of one species concentration depends on the concentration of the other species. It is built from the Jacobian matrix *J*_*f*_(*x*^∗^) of *f* evaluated at a point *x*^∗^, such that the non-zero entries of *J*_*f*_(*x*^∗^) correspond to directed edges of the graph and the signs of the entries are edge labels. Soulé proved that the existence of a positive feedback loop in the interaction graph is a necessary condition for *f*(*x*) to have multiple zeros. In other words, it is a necessary condition for multi-stationarity to exist in the ODE system (1).

Soulé’s approach is often not useful for many reaction networks, such as enzymatic signaling networks, because the edge labels are not constant, but depend on the concentrations of the species, that is, for some concentrations a label might be positive, for others it might be negative. A refinement of Soulé’s work is based on the so-called *directed species-reaction graph* (DSR-graph) [13,33-35]. If *f* in (1) is obtained from a reaction network, then it decomposes in the form

(2) $$ \dot{x}= f(x) = A v(x),  $$

where *A* is the stoichiometric matrix of the network and *v*(*x*) the vector of reaction rates. The DSR-graph uses this particular structure.

The DSR-graph is a bipartite graph with nodes labeled by the species and the reactions of the reaction network. Labeled directed edges from species nodes to reaction nodes and from reaction nodes to species nodes are derived from the vector of reaction rates *v*(*x*) and the stoichiometric matrix *A*, respectively. Compared to the interaction graph, the DSR-graph makes use of the explicit decomposition (2) of *f*.

It has been shown that the existence of positive feedback loops in the DSR-graph is a necessary condition for the system (2) to admit multi-stationarity [34].

Based on these results it has become standard to highlight positive feedback loops in multi-stationary reaction networks, eg. [1,2]. The loops are typically found using intuitive reasoning that might overlook the existence of other relevant positive feedback loops or might select positive feedback loops that are not related to the existence of multi-stationarity. Here we provide a method, based on theoretical considerations, to classify *all* positive feedback loops of a multi-stationary network into those that are related to the observed multi-stationarity and those that are not. In other words, we determine the positive feedback loops that when they all are broken, multi-stationarity disappears.

The question needs to be understood in the context of the whole network and not in isolation: a particular positive feedback loop that is responsible for multi-stationarity in one network might appear in another network that cannot have multiple steady states.

We present an automated procedure to determine the positive feedback loops that contribute to multi-stationarity. The procedure is based on various ideas from previous work by us and others. In particular, it builds on the *injectivity* property applied to an ODE system of the form (2), as described in [13]. The procedure can be applied to any network, provided that the components of the vector *v*(*x*) are strictly monotone with respect to all variables. This is a mild assumption that is fulfilled for typical kinetics such as mass-action kinetics and Michaelis-Menten kinetics.

In Methods, we introduce the necessary background material. As part of this we explain why positive feedback loops are necessary for multi-stationarity and how this relates to the DSR-graph. In Results, we present the automated procedure and how it selects the relevant positive feedback loops out of all positive feedback loops. We further apply the procedure to examples of multi-stationary reaction networks involved in cell signaling. We also consider the networks in the Biomodels database [36] and apply the procedure to all non-injective networks (injective networks cannot be multi-stationary, see below). This provides an overview of the landscape of relevant positive feedback loops occurring in documented reaction networks.

## Methods

In this section we introduce the different ideas we need to construct the automated procedure. We use the formalism of Chemical Reaction Network Theory (CRNT) [37]. An ODE system is built from a set of reactions and reaction rates, which we explain in the section below.

### Reaction networks

**Figure 1:**
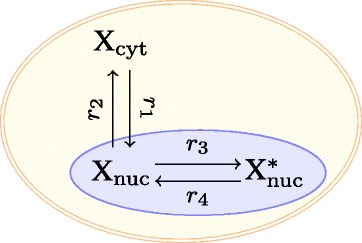
Main example. The reaction network used in [1] as a toy model to model the onset of mitosis. Here X is the complex Cdk1-cyclin B1 formed by the cyclin dependent kinase Cdk1 and the mitotic cyclin B1, “cyt” indicates that the species is in the cytoplasm, “nuc” that it is in the nucleus, and X ^∗^ is phosphorylated CdC1-cyclin B1. Phosphorylation of Cdk1-cyclin B1 only takes place in the cell nucleus.

A *reaction network*, or simply a *network*, consists of a set of species {*X*_1_,…,*X*_*n*_} and a set of reactions of the form:

(3) $$ r_{j}\colon \sum_{i=1}^{n} \alpha_{ij} X_{i} \rightarrow \sum_{i=1}^{n}\beta_{ij} X_{i}, \qquad j=1,\dots,m  $$

where *α*_*ij*_,*β*_*ij*_ are nonnegative integers, called the stoichiometric coefficients of the reactants and the products, respectively. As a running example we use the network in Figure 1. It has three species, X _cyt_, X _nuc_, X$^{*}_{\text {nuc}}$, which are different forms of the Cdk1-cyclin B1 complex, and four reactions [1].

We denote the concentration of the species *X*_1_,…,*X*_*n*_ by lower-case letters *x*_1_,…,*x*_*n*_. The evolution of the species concentrations with respect to time is modelled as an ODE system in the following way. We let *A*=(*a*_*ij*_) be the stoichiometric matrix of the network:

$$a_{ij} = \beta_{ij}-\alpha_{ij}, $$

that is, the (*i*,*j*)-th entry encodes the net stoichiometric coefficient of species *X*_*i*_ in reaction *r*_*j*_. The vector (*a*_1*j*_,…,*a*_*nj*_) is called the *reaction vector* of reaction *r*_*j*_.

The rate of reaction *r*_*j*_ is a function $v_{j}\colon \Omega _{v} \rightarrow \mathbb {R}_{\geq 0}$, where $\mathbb {R}_{>0}^{n}\subseteq \Omega _{v} \subseteq \mathbb {R}^{n}_{\geq 0}$ and *Ω*_*v*_ is the set of possible species concentrations. A typical choice of *v*=(*v*_1_,…,*v*_*m*_) is *mass-action kinetics*. In this case

$$ v_{j}(x)=\kappa_{j} \,x_{1}^{\alpha_{1j}}\cdot \dots \cdot x_{n}^{\alpha_{nj}},\quad x\in\Omega_{v}, $$

where *κ*_*j*_ is a positive reaction rate constant and 0^0^=1 by convention. Putting the pieces together provides a model for the evolution of the species concentrations over time:

(4) $$ \dot{x} = Av(x),\qquad x\in \Omega_{v}.  $$

Returning to Figure 1, we let *x*_1_,*x*_2_,*x*_3_ be the concentrations of X _cyt_, X _nuc_, X$^{*}_{\text {nuc}}$, respectively. Following [1], one model of the network is:

(5) $$\begin{array}{*{20}l} \dot{x}_{1} & = -\kappa_{1} x_{1} + \kappa_{2} x_{2}  \\ \dot{x}_{2}& = \kappa_{1} x_{1} - \kappa_{2} x_{2} - \frac{x_{2} (x_{2}+x_{3})^{4} }{K^{4}+(x_{2}+x_{3})^{4}} + \kappa_{4} x_{3} \\ \dot{x}_{3} & = \frac{x_{2} (x_{2}+x_{3})^{4} }{K^{4}+(x_{2} +x_{3})^{4}} - \kappa_{4} x_{3},  \end{array} $$

where *κ*_1_,…,*κ*_4_,*K*>0 are parameters. It takes the form (4) with

(6) $$ A=\left(\begin{array}{rrrr} -1 & 1 & 0 & 0 \\ 1 & -1 & -1 & 1 \\ 0 & 0 & 1 & -1 \end{array} \right),  $$

(7) $$ v(x)=\left(\kappa_{1} x_{1},\kappa_{2} x_{2},\frac{x_{2} (x_{2}+x_{3})^{4} }{K^{4}+(x_{2}+x_{3})^{4}},\kappa_{4} x_{3}\right),  $$

and $\Omega _{v}=\mathbb {R}^{n}_{\ge 0}$. Observe that the phosphorylation reaction $\mathrm {X}_{\text {nuc}} \rightarrow \mathrm {X}^{*}_{\text {nuc}}$ has a reaction rate that depends on both the concentration of the reactant X _nuc_ and the concentration of the product X$^{*}_{\text {nuc}}$. We also consider an alternative model in which the rate of X _nuc_ phosphorylation depends on *x*_2_ only:

(8) $$ v(x)=\left(\kappa_{1} x_{1},\kappa_{2} x_{2}, \frac{{x_{2}^{5}} }{K^{4}+{x_{2}^{4}}},\kappa_{4} x_{3}\right).  $$

This alternative model is also consistent with the set of reactions in Figure 1, but the third reaction is now independent of the amount of X$^{*}_{\text {nuc}}$.

### Multi-stationarity

In general the trajectory of the ODE system (4) determined by an initial positive condition is confined to a particular subset of $\mathbb {R}_{\geq 0}^{n}$, called a *stoichiometric compatibility class* [37]. For instance, in the running example, the quantity *T*(*x*)=*x*_1_+*x*_2_+*x*_3_ is conserved through time and determined by its value at time 0. This equation (called a *conservation law*), and the value it takes, characterises the stoichiometric compatibility class. Two trajectories with different initial conditions but with the same value of *T*(*x*) are confined to the same stoichiometric compatibility class.

The stoichiometric compatibility classes are defined as (see [37])

$$ \mathcal C_{0}= \left(x_{0}+ \text{im}(A)\right) \cap\mathbb{R}_{\geq 0}, $$

where *x*_0_=*x*(0) in $\mathbb {R}_{>0}$ is the initial condition and im(*A*) denotes the image of *A*. That is, the trajectories are restricted to the space spanned by the reaction vectors. Any trajectory that starts in the interior of $\mathcal C_{0}$, stays there, but might be attracted towards the boundary.

A reaction network is said to be *multi-stationary* if there exist two distinct positive steady states in a stoichiometric compatibility class (but not necessarily in all classes) [37]. Equivalently, if there exist distinct positive $x,y\in \mathbb {R}^{n}_{>0}$ such that *Av*(*x*)=*Av*(*y*)=0 and *x*−*y*∈im(*A*). A network with one positive steady state and one steady state at the boundary is therefore not multi-stationary in this terminology.

The reaction network in Figure 1 is multi-stationary for some choice of parameters with the rate vector in (7) [1], but not with the rate vector in (8) for all choice of parameter values (which will be shown later).

### Influence matrix

The concept of a positive feedback loop is associated with structural network properties and qualitative features of the reaction rates. Therefore, we assume some regularity on the reaction rates which we will encode into an abstract symbolic matrix, called the *influence matrix* [13] (see also [35]). A feedback loop does not depend on specific parameters or the specific functional form of the reaction rates.

To proceed, we assume that the function *v*_*j*_(*x*) is strictly monotone in each variable *x*_*i*_ and define the influence matrix *Z*=(*z*_*ij*_) as

(9) $$  z_{ij} = \begin{cases} \gamma_{ij} & \textrm{if }v_{j}(x)\textrm{increases in }x_{i}\\ -\gamma_{ij} & \textrm{if }v_{j}(x)\textrm{decreases in }x_{i} \\ 0 & \textrm{if }v_{j}(x)\textrm{is constant in }x_{i}, \end{cases}  $$

where *γ*_*ij*_ are symbolic variables.

The influence matrices associated with the two reaction rate vectors in (7) and (8) are given by

(10) $$ Z_{1}=\left(\begin{array}{cccc} \gamma_{1,1} & 0 & 0 & 0 \\ 0 & \gamma_{2,2} & \gamma_{2,3} & 0 \\ 0 & 0 & \gamma_{3,3} & \gamma_{3,4} \end{array} \right),  $$

and

(11) $$ Z_{2}=\left(\begin{array}{cccc} \gamma_{1,1} & 0 & 0 & 0 \\ 0 & \gamma_{2,2} & \gamma_{2,3} & 0 \\ 0 & 0 & 0& \gamma_{3,4} \end{array} \right),  $$

respectively. In (10) and (11), all influences are zero or positive.

In the following sections we will develop the graphical framework that we use to find the relevant positive feedback loops. It builds on the DSR-graph [13] (see also [34,35] for alternative definitions of the DSR-graph). Subsequently, we define circuits and nuclei based on Soulé’s work [30].

### DSR-graph

We define the DSR-graph as a labelled bipartite directed graph with node set {*X*_1_,…,*X*_*n*_,*r*_1_,…,*r*_*m*_} and such that:

- There is an edge from *X*_*i*_ to *r*_*j*_ with label *z*_*ij*_ if *z*_*ij*_≠0.
- There is an edge from *r*_*j*_ to *X*_*i*_ with label *a*_*ij*_ if *a*_*ij*_≠0.

**Figure 2:**
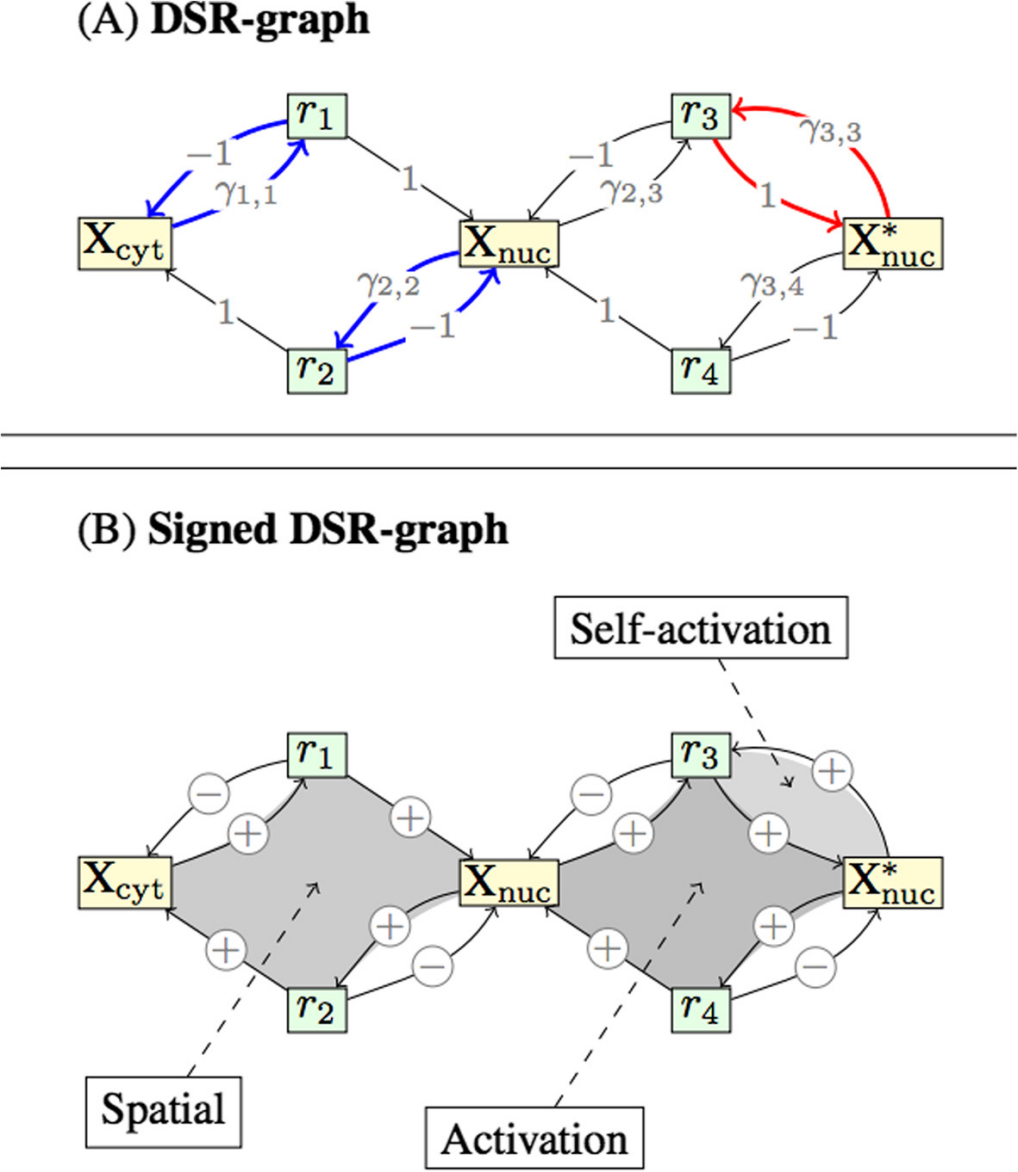
DSR-graphs of the running example.**(A)** The DSR-graph. There are two 4-nuclei corresponding to negative terms in the polynomial $p_{A,Z_{1}}$: each of them consists of the red circuit combined with one of the two blue circuits. Of these, the only positive feedback loop is the red circuit, which is responsible for the observed multi-stationarity. **(B)** There are three positive feedback loops in the graph, marked with shades of grey. Only the self-activation feedback loop (red circuit in (A)) is associated a term in the polynomial $p_{A,Z_{1}}$, see (14). Hence the other two positive feedback loops are not relevant for the observed multi-stationarity.

We refer to the *signed DSR-graph* as the graph identical to the DSR-graph given by (a)-(b), but with the labels replaced by their signs. The (signed) DSR-graph of the running example with *A* as in (6) and *Z* as in (10) is shown in Figure 2. The (signed) DSR-graph with *Z* as in (11) is identical to that in Figure 2, with the edge from X$^{*}_{\text {nuc}}$ to *r*_3_ removed.

### Circuits and nuclei

The positive feedback loops of the DSR-graph are instances of *circuits*, cycles of directed edges in a graph. Formally, a circuit in a graph *G* is a sequence of distinct nodes *i*_1_,…,*i*_*q*_ such that there is a directed edge from *i*_*k*_ to *i*_*k*+1_ for all *k*≤*q*−1 and one from *i*_*q*_ to *i*_1_. A circuit must involve at least one edge. The label of a circuit *C*, denoted *ℓ*(*C*), is the product of the labels of the edges in the circuit. Two circuits are disjoint if they do not have any common nodes. Three circuits are highlighted in Figure 2(A) (two blue and one red), each involving two nodes.

A circuit with positive label is a *positive feedback loop*, and similarly, a circuit with negative label is a negative feedback loop. The three positive feedback loops of the running example are shaded in Figure 2B. They correspond to shuttling of the complex between the nucleus and the cytoplasm, activation and deactivation of X _nuc_, and self-activation of X _nuc_ (the rate of reaction *r*_3_ increases with *x*_3_, that is, the production of X$^{*}_{\text {nuc}}$ increases with the amount of X$^{*}_{\text {nuc}}$).

A *k*-nucleus is a collection of disjoint circuits which involves *k* nodes [30]. The label *ℓ*(*D*) of a *k*-nucleus *D* is the product of the labels of the edges in the nucleus. Let *a*_1_,*a*_2_ be the number of circuits in the nucleus that have odd (resp. even) number of species nodes and let *a*=*a*_1_+*a*_2_. The sign of a *k*-nucleus is defined as *σ*(*D*)=(−1)^*a*^_2_. That is, if *D*=*C*_1_∪⋯∪*C*_*a*_ is a disjoint union of circuits, then

(12) $$ \sigma(D)\ell(D) =(-1)^{a_{2}} \prod_{i=1}^{a} \ell(C_{i}).  $$

In the DSR-graph, any circuit involves an equal number of species and reaction nodes and, hence, an even number of edges. We will consider nuclei with *k*=2*s* edges, where *s* is the rank of the matrix *A*. The reason for considering *k*=2*s* will become clear in the Section ‘The polynomial and circuits’. Let *D*=*C*_1_∪⋯∪*C*_*a*_ be a 2*s*-nucleus of the DSR-graph. If none of the circuits are positive feedback loops, then the sign of *σ*(*D*)*ℓ*(*D*) is (−1)^*s*^. Indeed, if all circuits have negative labels, that is, *ℓ*(*C*_*i*_) has negative sign for all *i*, then

$$\text{sign}(\sigma(D)\ell(D)) = (-1)^{a_{2}+a} = (-1)^{a_{1}+2a_{2}} = (-1)^{a_{1}}. $$

Because *D* is a 2*s*-nucleus, it contains *s* species nodes. Let *n*_*i*_ be the number of species nodes in circuit *C*_*i*_, such that *s*=*n*_1_+⋯+*n*_*a*_. We have that *n*_*i*_ is odd for *a*_1_ of the circuits and even for *a*_2_ of the circuits. Therefore, $(-1)^{s} = (-1)^{n_{1}+\dots +n_{a}} = (-1)^{a_{1}},$ and

(13) $$ \text{sign}(\sigma(D)\ell(D))=(-1)^{s}  $$

if there are no positive feedback loops in *D*. This result is also in [35], where it is stated using a different terminology.

We next we introduce the concept of injectivity and then link it to circuits and positive feedback loops in a separate section.

### Injectivity

In this section we study the injectivity of the function *x*↦*Av*(*x*), $x\in \mathcal C_{0}$ (4). The injectivity property has been discussed in different contexts, see for example [12,13,15,35,38,39]. We use here the approach given in [13].

In the next section we link this injectivity property for all positive stoichiometric compatibility classes to the non-existence of positive feedback loops in the DSR-graph. With other words, if all feedback loops are negative then the function is injective on all positive stoichiometric compatibility classes. In particular, there cannot exist two distinct points $x,y\in \mathbb {R}^{n}_{>0}$ in the same stoichiometric compatibility class such that *Av*(*x*)=*Av*(*y*)=0, that is, the network cannot be multi-stationary.

To decide whether the function *Av*(*x*) is injective on all positive stoichiometric compatibility classes for any *v*(*x*) with given influence matrix *Z*, we rely on a method previously developed by us [12,13]. We will now explain this method.

Given a stoichiometric matrix *A* and an influence matrix *Z*, we define a polynomial *p*_*A*,*Z*_ of degree *s*=rank(*A*), in as many variables as there are non-zero entries of *Z*. For this, let *M*=*AZ*^*t*^ and let {*ω*^1^,…,*ω*^*d*^} be a basis of im(*A*)^⊥^, which we assume to be Gauss-reduced. Further, let *i*_1_,…,*i*_*d*_ be the indices of the first non-zero entries of *ω*^1^,…,*ω*^*d*^, respectively. We define a symbolic *n*×*n* matrix, $\widetilde {M}$, by replacing the *i*_*j*_-th row of *M* with *ω*^*j*^ (cf. [13], Section 5). The polynomial *p*_*A*,*Z*_ is defined as

$$p_{A,Z}=\det(\widetilde{M}), $$

which can be written as a sum of terms depending on the variables *γ*_*ij*_, by expanding the determinant. Each non-zero term is of the form $c\prod _{k=1}^{s} \gamma _{i_{k}j_{k}}$ where *c* is a coefficient (positive or negative) and all *i*_*k*_, respectively *j*_*k*_, are distinct.

It is a result of [12,13] that if *p*_*A*,*Z*_ is not identically zero and all non-zero coefficients of *p*_*A*,*Z*_ have the same sign, then the function *Av*(*x*) is injective on each positive stoichiometric compatibility class and, hence, the network cannot be multi-stationary. As a consequence, *p*_*A*,*Z*_ has coefficients of opposite sign whenever the network is multi-stationary. If the coefficients do not have the same sign, then the network might be multi-stationary, but it cannot be concluded from the test.

Consider the matrix *A* given in (6) and *Z*_1_ in (10). We choose {(1,1,1)} as a basis of im(*A*)^⊥^ and obtain

(14) $$\begin{array}{*{20}l} p_{A,Z_{1}} & = -\gamma_{2,2}\gamma_{3,3}- \gamma_{1,1}\gamma_{3,3}  \\ & \gamma_{2,2} \gamma_{3,4} + \gamma_{1,1}\gamma_{2,3} + \gamma_{1,1}\gamma_{3,4}.  \end{array} $$

There are both positive and negative terms, hence multi-stationarity cannot be excluded. For *Z*_2_ in (11), the polynomial $p_{A,Z_{2}}$ is obtained from (14) by setting *γ*_3,3_=0,

(15) $$\begin{array}{*{20}l} p_{A,Z_{2}} & = \gamma_{2,2} \gamma_{3,4} + \gamma_{1,1}\gamma_{2,3} + \gamma_{1,1}\gamma_{3,4}.  \end{array} $$

In this case all terms have the same sign and thus, the network cannot be multi-stationary. This holds for any choice of rate functions with influence matrix *Z*_2_.

### The polynomial and circuits

Finally, we link injectivity and the polynomial *p*_*A*,*Z*_ to positive feedback loops. It is shown in [13] that each term of the polynomial *p*_*A*,*Z*_ can be identified with a *collection* of disjoint unions of circuits in the DSR-graph *G*. Specifically, given subsets *I*,*J*⊆{1,…,*n*} of cardinality *s*, let *D*_*s*_(*I*,*J*) be the set of 2*s*-nuclei of *G* with node set {*X*_*i*_| *i*∈*I*}∪{*r*_*j*_| *j*∈*J*}. Then

(16) $$ p_{A,Z} = \sum_{I,J\subseteq \{1,\dots,n\}} \sum_{D \in D_{s}(I,J)} \sigma(D)\ell(D),  $$

where the sets *I*,*J* in the sum have cardinality *s* (cf. [13], Section 11).

Let *D*∈*D*_*s*_(*I*,*J*) be a 2*s*-nucleus. If none of the circuits of the nucleus are positive feedback loops then the sign of *σ*(*D*)*ℓ*(*D*) is (−1)^*s*^, see Section ‘Circuits and nuclei’ and (13). Hence if the DSR-graph contains a positive feedback loop in a 2*s*-nucleus then the sign of *σ*(*D*)*ℓ*(*D*) must be (−1)^*s*+1^. This observation will be crucial for the automated procedure.

## Results

We begin by summarising the key ingredients described in Methods that lead to the proposed automated procedure. The (computable) polynomial *p*_*A*,*Z*_ might have positive and negative terms. On one hand, each term in the polynomial corresponds to a collection of nuclei, as described in the Section ‘The polynomial and circuits’. Each of these nuclei consists of circuits, some of which might be positive feedback loops. A term of sign (−1)^*s*+1^ in the polynomial necessarily corresponds to a collection of nuclei involving at least one positive feedback loop, see Section ‘The polynomial and circuits’.

On the other hand, the network can only be multi-stationary if the polynomial *p*_*A*,*Z*_ has terms of opposite sign. Consequently, if the network is multi-stationary, then some terms of *p*_*A*,*Z*_ have the sign (−1)^*s*+1^. Therefore we conclude that (i) the network must contain positive feedback loops in order to be multi-stationary and (ii) the positive feedback loops underlying multi-stationarity can be found by considering the terms with sign (−1)^*s*+1^ in the polynomial. From these terms we identify the associated nuclei and extract the positive feedback loops in these nuclei.

It is an empirical observation that in most realistic or real reaction networks, the predominant sign of the coefficients of *p*_*A*,*Z*_ is (−1)^*s*^. Therefore, the number of terms to inspect, that is, the number of terms with sign (−1)^*s*+1^, is usually low. For this reason, we call the sign (−1)^*s*+1^ the ‘wrong sign’.

Based on these considerations, we develop a procedure to extract the positive feedback loops that correspond to terms with the wrong sign in *p*_*A*,*Z*_. The procedure is based on the following steps. For a non-zero term with the wrong sign, say

(17) $$ (-1)^{s+1}c\, \gamma_{i_{1},j_{1}}\dots \gamma_{i_{s},j_{s}}  $$

(*c* is positive) consider the following edges from species to reactions

$$X_{i_{k}}\xrightarrow{\pm \gamma_{i_{k},j_{k}}} r_{j_{k}}. $$

The 2*s*-nuclei corresponding to the term (17) must contain these edges. Therefore, we add to these edges all possible choices of *s* edges from reactions $\{r_{j_{1}},\dots,r_{j_{s}}\}$ to species $\{X_{i_{1}},\dots,X_{i_{s}}\}$ such that the resulting graph is a 2*s*-nucleus. We keep only the nuclei *D* for which the sign of *σ*(*D*)*ℓ*(*D*) is (−1)^*s*+1^. The positive feedback loops in these nuclei are those that *do* contribute to the existence of multiple steady states. Indeed, if all these loops are broken, then the network cannot be multi-stationary because the polynomial will then only have terms of sign (−1)^*s*^. We find these loops in the signed DSR-graph.

For example, consider the polynomial $p_{A,Z_{1}}$ in (14) and the DSR-graph shown in Figure 2. In this case, the rank of *A* is *s*=2, and hence we focus on the negative terms since (−1)^*s*+1^=−1. These are *γ*_2,2_*γ*_3,3_ and *γ*_1,1_*γ*_3,3_. The corresponding 4-nuclei are depicted in Figure 2(A): there are two 4-nuclei obtained as the union of the red circuit and one of the two blue circuits. The only positive feedback loop that appears is therefore the self-activation positive feedback loop, and this is the only loop that is related to the observed multi-stationarity. The other two positive feedback loops (termed the spatial and the activation loop, respectively in Figure 2(B)) are therefore not relevant for the observed multi-stationarity.

We next state the automated procedure.

### Automated procedure

The procedure to select positive feedback loops that contribute to multi-stationarity, applies to *any* reaction network defined as in (4), which fulfils the monotonicity criteria (9). This criterion states that the reaction rates *v*(*x*) must be strictly monotone in each variable *x*_*j*_, otherwise the influence matrix in not uniquely defined.

The procedure consists of the following steps.

1. For a network with stoichiometric matrix *A* of rank *s* and influence matrix *Z*, compute *p*_*A*,*Z*_ and select the terms with sign (−1)^*s*+1^.
2. Construct the DSR-graph. For each selected term of *p*_*A*,*Z*_ with the wrong sign, determine the corresponding 2*s*-nuclei of the DSR-graph that have the wrong sign.
3. For each of the nuclei, select the connected components that form positive feedback loops.

These steps have been implemented in Maple. The Maple script is provided as Additional file 1. The procedure might fail for practical reasons (such as lack of computational memory) if the number of species and reactions is too big. In our experience, this number depends heavily on the sparsity of the influence matrix [12].

It is worth emphasising that the procedure is meaningful only if we know that the input network is multi-stationary. We might apply the procedure to a non-multi-stationary network and obtain a list of positive feedback loops, but these loops will lack proper interpretation.

### Examples

We have applied the procedure to find positive feedback loops that are responsible for multi-stationarity in several reaction networks. These examples are also shown in the Additional file 1, together with some other systems such as the three-site phosphorylation system.

#### Ring1B/Bmi1 ubiquitination system

We consider an ODE model of histone H2A ubiquitination that involves the E3 ligases Ring1B and Bmi1 [3]. When degradation of species is not taken into account, the model has 10 species and 15 reactions. We let B and B$^{d}_{\textit {ub}}$ denote the protein Bmi1 in isolation and targeted for degradation by ubiquitination, respectively. The protein Ring1B is denoted by R, and R _*ub*_, R$_{\textit {ub}}^{a}$, R$^{d}_{\textit {ub}}$ denote three different forms of self-ubiquitinated R, with R$^{d}_{\textit {ub}}$ being the form targeted for degradation. Bmi1 and Ring1B form a complex Z, that also might be ubiquitinated, Z _*ub*_. Finally, Ring1B (either alone or in the complex Z) is responsible for the ubiquitination of the histone H2A, with states H, H _*ub*_.

The reactions describing the mechanism are [3]:

$$\begin{array}{*{20}l} \mathrm{B} & {\overset{r_{1}}{\underset{{r_{2}}}{\rightleftharpoons}}}\mathrm{B}_{ub}^{d} & \mathrm{R} & {\overset{r_{3}}{\underset{{r_{4}}}{\rightleftharpoons}}}\mathrm{R}_{ub}^{d} & \mathrm{B}+\mathrm{R} & {\overset{r_{5}}{\underset{{r_{6}}}{\rightleftharpoons}}}\mathrm{Z} \\ \mathrm{Z} & {\overset{r_{7}}{\underset{{r_{8}}}{\rightleftharpoons}}}\mathrm{Z}_{ub} & \mathrm{Z}_{ub} & {\overset{r_{9}}{\underset{{r_{10}}}{\rightleftharpoons}}}\mathrm{B}+\mathrm{R}_{ub}^{a} & \mathrm{R} & {\overset{r_{11}}{\underset{{r_{12}}}{\rightleftharpoons}}}\mathrm{R}_{ub} \\ \mathrm{R}_{ub}^{a} & \xrightarrow{r_{13}}\mathrm{R} & \mathrm{H} & {\overset{r_{14}}{\underset{{r_{15}}}{\rightleftharpoons}}} \mathrm{H}_{ub}. \end{array} $$

We let *x*_1_,…,*x*_10_ denote the concentrations of B, B$_{\textit {ub}}^{d}$, R, R$_{\textit {ub}}^{d}$, R _*ub*_, R$_{\textit {ub}}^{a}$, Z, Z _*ub*_, H, H _*ub*_, respectively. The associated reaction rates are [3]:

$$\begin{array}{*{20}l}  v_{1} &= \kappa_{1}x_{1} & v_{2} &= \kappa_{2}x_{2} \\  v_{3} & =\kappa_{3}x_{3} & v_{4} &=\kappa_{4}x_{4} \\  v_{5} &=\kappa_{5}x_{1}x_{3} & v_{6} & =\kappa_{6}x_{7} \\  v_{7} &=x_{7}(\kappa_{7}x_{7}+\kappa_{8}x_{8}) & v_{8} &= \kappa_{9}x_{8} /(\kappa_{10}+x_{8}) \\  v_{9} &= \kappa_{11}x_{8} & v_{10} &= \kappa_{12}x_{1} x_{6} \\  v_{11} &=\kappa_{13}{x_{3}^{2}}+\kappa_{14}x_{3}x_{5} & v_{12} &=\kappa_{14}x_{5} \\ v_{13} &=\kappa_{15}x_{6} & v_{15} &=\kappa_{19}x_{10}, \\ v_{14} &=x_{9} (\kappa_{16}x_{5}+ \kappa_{17} x_{8}+ \kappa_{18}x_{6}), \end{array} $$

where *κ*_*i*_>0 are constants.

**Figure 3:**
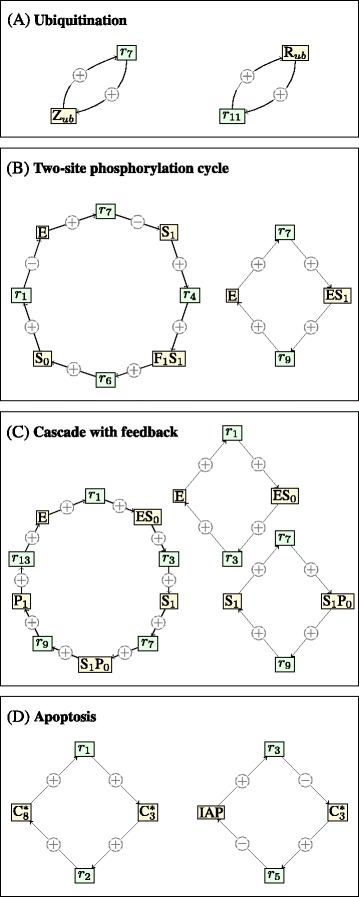
Examples.**(A)** For the ubiquitination system two positive feedback loops are found. The loops correspond to self-ubiquitination of Z and R, respectively. **(B)** There are two positive feedback loops. The right loop corresponds to the Michaelis-Menten mechanism involving the two species E and ES_1_. The left loop has four species nodes. The substrates S_0_ and S_1_ compete for the same kinase E in a way that enhances the production of both substrates: increasing S_0_, decreases the amount of E (reaction *r*_1_) which decreases the rate of reaction *r*_7_, which in turn increases the amount of S_1_. **(C)** Of the three positive feedback loops that are found, two correspond to the Michaelis-Menten mechanism (right side). One involves the kinase E and the complex ES_0_. The second is similar, involving the kinase S_1_ of the second layer and the complex S_1_P_0_. The left loop has five species nodes and illustrates P_1_-activation of the kinase E. **(D)** The apoptosis system has two loops. The left loop occurs because C 8^∗^ in reaction *r*_1_ increases the amount of C 3^∗^, which in turn increases the amount of C 8^∗^ via reaction *r*_2_.

Self-ubiquitination of B is taken into account in the rate functions *v*_7_ and *v*_11_ for reactions *r*_7_ and *r*_11_, respectively. These incorporate a positive influence from the reaction products. With these specific rate functions, the system is multi-stationary for some values of the reaction rate constants [3]. We apply the automated procedure to find positive feedback loops that are responsible for multi-stationarity and obtain the circuits depicted in Figure 3(A). In [3], it is postulated that self-ubiquitination of Z and R are crucial steps for the emergence of multiple steady states, and we confirm the statement here.

#### Phosphorylation systems

We have analysed different networks of signal transmission based on phosphorylation. We consider models for sequentially distributed phosphorylation and dephosphorylation cycles and some modifications of these, see e.g. [10,40].

We consider first a two-site sequential phosphorylation cycle for a substrate S, where phosphorylation of the two sites is catalysed distributively by a kinase E, and dephosphorylation of the two sites uses different phosphatases F_1_, F_2_. Assuming a Michaelis-Menten mechanism, the reaction network consists of the following reactions:

$$\begin{array}{*{20}l} \mathrm{E}+ \mathrm{S}_{0} {\overset{r_{1}}{\underset{{r_{2}}}{\rightleftharpoons}}} \text{ES}_{0}\xrightarrow{r_{3}} \mathrm{E} + \mathrm{S}_{1} {\overset{r_{7}}{\underset{{r_{8}}}{\rightleftharpoons}}} \text{ES}_{1} \xrightarrow{r_{9}} \mathrm{E} + \mathrm{S}_{2} \\ \mathrm{F}_{1} + \mathrm{S}_{1} {\overset{r_{4}}{\underset{{r_{5}}}{\rightleftharpoons}}} \mathrm{F}_{1}\mathrm{S}_{1} \xrightarrow{r_{6}} \mathrm{F}_{1} + \mathrm{S}_{0} \\ \mathrm{F}_{2}+ \mathrm{S}_{2} {\overset{r_{10}}{\underset{{r_{11}}}{\rightleftharpoons}}} \mathrm{F}_{2}\mathrm{S}_{2} \xrightarrow{r_{12}} \mathrm{F}_{2} + \mathrm{S}_{1} \end{array} $$

In S_0_, S_1_, S_2_ the subindex denotes the number of phosphorylated sites. With mass-action kinetics, this system is multi-stationary for some choice of reaction rate constants [2,10]. However, the positive feedback loops that can account for the observed multi-stationarity are not trivial.

We apply the automated procedure and obtain the positive feedback loops given in Figure 3(B). The first of the two loops has two edges with negative labels. It implies that S_0_ and S_1_ enhance their own production. Indeed, because S_0_ and S_1_ both compete for the same kinase, an increase in S_0_ increases the rate of reaction *r*_1_, which in turn lowers the amount of available enzyme E. This implies that reaction *r*_7_ slows down and hence S_1_ is consumed at a slower rate. The circuit closes through the reactions *r*_4_ and *r*_6_, which shows that an increase in S_1_ implies an increase in S_0_.

These type of loops are recurrent in phosphorylation motifs. It is worth emphasising that the loops do not have independent meaning outside the network. Another network with the same positive feedback loop might not be multi-stationary. For example, the second loop of Figure 3(B) also occurs in the Michaelis-Menten mechanism $\mathrm {S}_{0}+\mathrm {E}\leftrightharpoons \text {ES}_{0}\rightarrow \mathrm {S}_{1}+\mathrm {E}$, but these reactions alone are not multi-stationary.

#### Signalling cascades

We consider a 2-layer cascade with an explicit positive feedback. The first layer is a phosphorylation cycle with kinase E, phosphatase F_1_, and phosphorylated and unphosphorylated substrate S_0_, S_1_. The second layer has kinase S_1_, phosphatase F_2_, and phosphorylated and unphosphorylated substrate P_0_, P_1_. Assuming a Michaelis-Menten mechanism, the reaction network consists of the following reactions:

$$\begin{array}{*{20}l} \mathrm{E}+ \mathrm{S}_{0} {\overset{r_{1}}{\underset{{r_{2}}}{\rightleftharpoons}}} \text{ES}_{0} \xrightarrow{r_{3}} \mathrm{E} + \mathrm{S}_{1} \\ \mathrm{F}_{1} + \mathrm{S}_{1} {\overset{r_{4}}{\underset{{r_{5}}}{\rightleftharpoons}}} \mathrm{F}_{1}\mathrm{S}_{1} \xrightarrow{r_{6}} \mathrm{F}_{1} + \mathrm{S}_{0} \\ \mathrm{S}_{1}+ \mathrm{P}_{0} {\overset{r_{7}}{\underset{{r_{8}}}{\rightleftharpoons}}} \mathrm{S}_{1}\mathrm{P}_{0} \xrightarrow{r_{9}} \mathrm{S}_{1} + \mathrm{P}_{1} \\ \mathrm{F}_{2} + \mathrm{P}_{1} {\overset{r_{10}}{\underset{{r_{11}}}{\rightleftharpoons}}} \mathrm{F}_{2}\mathrm{P}_{1} \xrightarrow{r_{12}} \mathrm{F}_{2} + \mathrm{P}_{0} \\ \mathrm{P}_{1} \xrightarrow{r_{13}} \mathrm{E}. \end{array} $$

This network is multi-stationary for some choice of reaction rate constants. The automated procedure finds three positive feedback loops, as shown in Figure 3(C). The first loop is expected and appears because the product of the second layer, P_1_, activates the kinase of the first layer, E. The other two loops are similar to those in Figure 3(C).

#### Apoptosis

We finally consider a basic model of caspase activation for apoptosis [9]:

$$\begin{array}{*{20}l} \mathrm{C}8^{*}+\mathrm{C}3 & \xrightarrow{r_{1}} \mathrm{C}8^{*}+\mathrm{C}3^{*} & \mathrm{C}8 &{\overset{r_{6}}{\underset{{r_{7}}}{\rightleftharpoons}}} 0\\ \mathrm{C}8+\mathrm{C}3^{*} & \xrightarrow{r_{2}} \mathrm{C}8^{*}+\mathrm{C}3^{*} & \mathrm{C}3 & {\overset{r_{8}}{\underset{{r_{9}}}{\rightleftharpoons}}}0\\ \mathrm{C}3^{*}+\text{IAP} & {\overset{r_{3}}{\underset{{r_{14}}}{\rightleftharpoons}}} \mathrm{Y} \xrightarrow{r_{4}} 0 & \text{IAP} & {\overset{r_{10}}{\underset{{r_{11}}}{\rightleftharpoons}}} 0 \\ \mathrm{C}3^{*}+\text{IAP} & \xrightarrow{r_{5}} \mathrm{C}3^{*}\xrightarrow{r_{13}} 0 & \mathrm{C}8^{*} & \xrightarrow{r_{12}}0. \end{array} $$

With mass-action kinetics, this network is multi-stationary for some choice of reaction rate constants [9] and has two relevant positive feedback loops, Figure 3(D). The second loop consists of two species, each with positive influence on a reaction rate, while at the same time, decreasing the amount of the other.

### Analysis of the Biomodels database

We investigated the models in the database PoCaB [41], which consists of 365 models from the publicly available database Biomodels [36] (see also the page http://www.ebi.ac.uk/biomodels-main/). The database PoCaB contains pre-computed stoichiometric matrices, mass-action exponent matrices, and kinetic data from the selected models.

In a previous paper [12] we extracted influence matrices of the reported kinetics. Of the 365 networks, 323 have strictly monotone kinetics such that the influence matrix is well defined. On these 323 networks we ran the injectivity method and ended up with a total of 64 non-injective networks, excluding 27 very large networks where the injectivity method failed (as described in [12]). Non-injectivity is a prerequisite for being multi-stationary.

**Figure 4:**
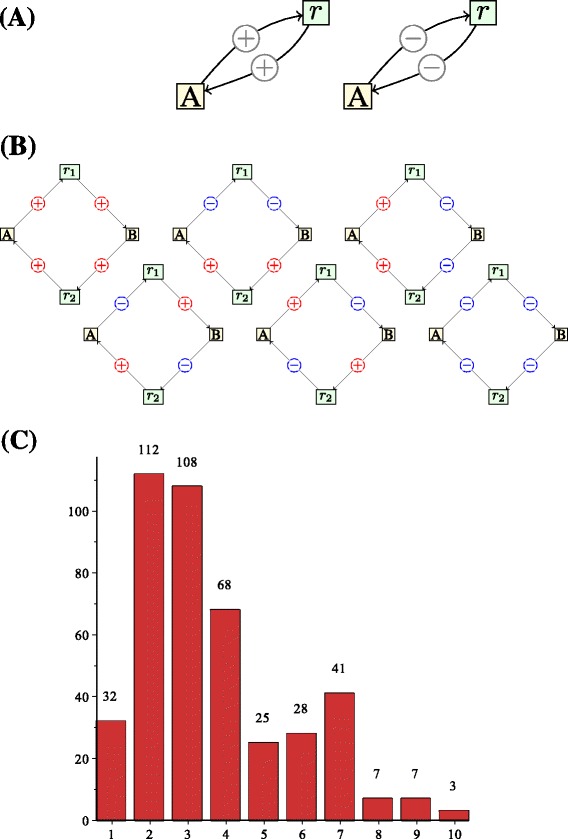
Biomodels database.**(A)** The positive feedback loops with one species. Among the 32 loops with one species, the frequencies are 19 and 13. **(B)** The positive feedback loops with 2 species. Among the 108 loops with 2 species, the frequencies are (from top left, row by row): 35, 16, 9, 16, 13, 23. **(C)** The histogram shows the size (number of species) distribution among the 341 positive feedback loops found in the 64 models.

We applied the automated procedure on the 64 networks to determine the positive feedback loops. We obtained a total of 341 different positive feedback loops with size distribution shown in Figure 4(C). Most loops involve only 2 or 3 species (112 and 108 loops out of 341, respectively). In Figure 4(A-B), we show the positive feedback loops involving one or two species and conclude that all possible types occur in the database. However, for two species, their frequencies vary from 9 to 35 (out of 108), indicating that the motifs are not equally represented in the database (Pearson’s chi-square test, *p*<0.0005). For one species, there appears to be no difference (*p*=0.28). We show in Table 1 the most frequent positive feedback loops for different number of species nodes.

**Table 1:** **Positive feedback loops**

**N**	**Motif**	**Frequency**
2	(−,−,−,−)	23/112 = 0.21
	(+,+,+,+)	35/112 = 0.31
3	(+,−,+,−,−,−)	19/108 = 0.18
	(+,+,+,+,+,+)	18/108 = 0.17
4	(+,+,−,+,−,+,−,−)	15/68 = 0.22
	(+,+,+,+,−,+,−,+)	15/68 = 0.22
	(+,+,+,+,+,+,+,+)	14/68 = 0.21
5	(+,+,+,+,+,+,−,+,−,+)	9/25 = 0.36
	(+,+,+,+,+,+,+,+,+,+)	7/25 = 0.28
6	(+,+,+,+,+,+,+,+,−,+,−,+)	6/28 = 0.21
	(+,+,+,+,+,+,+,+,+,+,+,+)	7/28 = 0.25
7	(+,+,+,+,+,+,+,+,+,+,−,+,−,+)	14/41 = 0.34

For N=2, …,7 species nodes, the most frequent (>15%) positive feedback loops for each *N* are shown, together with their frequencies. At most four negative labels occur. Each cycle starts at a reaction node and the odd (even) labels correspond to reaction (species) nodes. Note that, for example, (-,-,+,+,+,+) and (+,+,-,-,+,+) are the same as they are permutations of each other.

## Discussion

We have presented an automated procedure to find the positive feedback loops in a multi-stationary network that are contributing to the multi-stationarity. The procedure relies on structural and qualitative features of the network together with a kinetics, and it is insensitive to the specific form of the rate functions. Only positive feedback loops that are contributing to the multi-stationarity of the network are reported, excluding those positive feedback loops that are not relevant.

Whether a loop is relevant or not, depends on the entire DSR-graph of the network (that is, the reactions and the influence matrix) and not just on the loop itself. In this sense, being a positive feedback loop related to an observed multi-stationarity, is a context or network dependent property. This fact has also been observed in [33-35]. In these papers, it is shown that the existence of a positive feedback loop fulfilling a certain extra condition is a requirement for multi-stationarity to occur. Specifically, the loop must either intersect another positive feedback loop in a specific way (called an *S-to-R intersection*) or fulfil a certain condition on the labels (called an *s-cycle*). The first possibility is network dependent. It is worth mentioning that there can be positive feedback loops that are s-cycles or that intersect another positive feedback loop in an S-to-R intersection without being relevant for the observed multi-stationarity. This is the case for most of the examples presented here. For example, the apoptosis network has another positive feedback loop, Y →*r*_14_→IAP→*r*_3_→Y (all edges are positive), which intersects the loop on the right side in Figure 3(D) in an S-to-R intersection.

The property of network dependence is further illustrated in Figure 2(A)-(B), where the procedure is applied to the reaction network in Figure 1 with influence matrix given by (10). The network models translocation and phosphorylation of a cyclin dependent kinase X in the onset of mitosis. Only one of the three positive feedback loops shown in Figure 2(B) can be associated with multi-stationarity in the model, namely the self-activating loop that stimulates the creation of phosphorylated X$^{*}_{\text {nuc}}$ in the nucleus. In [1], it is argued by different means than in this paper, that the spatial redistribution of the cyclin dependent kinase is important for creating the observed bistability. In contrast, our results suggest that the observed bistability is due to the self-activation loop of the phosphorylated complex in the nucleus.

The presented procedure cannot establish whether a reaction network is multi-stationary or not. Other means will here be required. If the procedure is applied to a reaction network that might or might not be multi-stationary, then the absence of positive feedback loops implies that the network cannot be multi-stationary, whereas the presence of positive feedback loops leaves the question open.

Whether a positive feedback loop found by the procedure is important in a biological or experimental context, is not addressed in this paper, but must be established in other ways, for example by experimental verification. A reaction network might only be multi-stationary for very specific choices of reaction rates, which might not be relevant in a particular experimental setting. As the procedure is parameter independent, any such verification and subsequent interpretation is beyond the scope of the procedure.

## Conclusions

It is well known that multistationarity requires the existence of a positive feedback loop. However, positive feedback loops are abundant in most biochemical reaction networks and it remained unclear how to select the positive feedback loops that could be accounted for underlying the observed multistationarity. In this work we have proposed an automatised method to determine the relevant positive feedback loops. The method is based on the mathematical arguments underlying the proof that positive feedback loops are necessary for multistationarity to occur. We have tested and illustrated the method with several reaction networks. An implementation of the method in Maple is provided as supplementary material.

## Additional file

Additional file 1
**Maple Script contains the automated procedure as well as examples.**
